# Large-scale production of bioactive recombinant human acidic fibroblast growth factor in transgenic silkworm cocoons

**DOI:** 10.1038/srep16323

**Published:** 2015-11-16

**Authors:** Feng Wang, Riyuan Wang, Yuancheng Wang, Ping Zhao, Qingyou Xia

**Affiliations:** 1 State Key Laboratory of Silkworm Genome Biology, Southwest University, Chongqing, China; 2 College of biotechnology, Southwest University, Chongqing, China

## Abstract

With an increasing clinical demand for functional therapeutic proteins every year, there is an increasing requirement for the massive production of bioactive recombinant human acidic fibroblast growth factor (r-haFGF). In this present study, we delicately explore a strategy for the mass production of r-haFGF protein with biological activity in the transgenic silkworm cocoons. The sequence-optimized haFGF was inserted into an enhanced sericin-1 expression system to generate the original transgenic silkworm strain, which was then further crossed with a PIG jumpstarter strain to achieve the remobilization of the expression cassette to a “safe harbor” locus in the genome for the efficient expression of r-haFGF. In consequence, the expression of r-haFGF protein in the mutant line achieved a 5.6-fold increase compared to the original strain. The high content of r-haFGF facilitated its purification and large-scald yields. Furthermore, the r-haFGF protein bioactively promoted the growth, proliferation and migration of NIH/3T3 cells, suggesting the r-haFGF protein possessed native mitogenic activity and the potential for wound healing. These results show that the silk gland of silkworm could be an efficient bioreactor strategy for recombinant production of bioactive haFGF in silkworm cocoons.

Human acidic fibroblast growth factor (haFGF) is a soluble heparin-binding protein that belongs to the fibroblast growth factor (FGF) family^1^. It harbors a molecular weight of 15.8 kDa with 140-amino-acid peptides and functions as a strong mitogen to stimulate the proliferation of many cell types of mesodermal, endodermal and neuroectodermal origin, and is accordingly thought to play an important role in regulating angiogenesis and neovascularization during development and wound repair. Thus the haFGF is highly valuable in research, diagnostics and angiogenic therapeutic applications. For example, haFGF is widely used clinically to promote the rehabilitation and reconstruction of blood vessels^2^,^3^, scalds, and wound healing and has therapeutic potential for cardiovascular disorders^4^. In addition, haFGF is also applied in cosmetics to maintain strong vitality in skin cells, equipoise pigment distribution and improve skin character^5^,^6^. However, the limited sources of haFGF make it difficult to meet demands for the large amounts required for both *in vivo* and *in vitro* applications. Thus there is an increasing interest in the cost effective and efficient production of recombinant FGFs for experimental and clinical applications. Over the past few decades, several expression systems including recombinant adeno-associated virus (rAAV)^7^, *E. coli*^8^,^9^,^10^, *Pichia pastoris*^11^, insect cell^12^, mammalian cell^13^, baculovirus^14^ and transgenic plant^15^ have attempted to produce recombinant FGFs. However, insolubility, pool yields, complicated processing and low bioactivity have severely limited their applications when meeting the marketable demands, especially, with an increasing number of applications for cell therapy and translational medicine.

Due to a breeding and domestication history of over 4000 years, the silk gland of silkworm *Bombyx mori* now possesses the huge ability to synthesize a large amount of silk proteins in its silk gland and secrete them as silk thread to build a cocoon, making it an ideal bioreactor mode organ for the mass production of valuable recombinant proteins^16^. The silk thread is composed of two types of silk protein, the major fibroin proteins, which are synthesized in the posterior silk gland cells and account for 70%–80% of the silk thread weight, and the hydrophilic glue sericin proteins, which are synthesized in the middle silk gland cells and account for 20%–30% of the silk thread weight^17^. The fibroin proteins consist of fibroin heavy chains (H-chains), fibroin light chains (L-chains), and fibrohexamerins with a molar ratio of 6:6:1^18^. The sericin proteins are mainly encoded by *sericin1* (*Ser1*), *sericin2* (*Ser2*), and *sericin3* (*Ser3*)^19^. Since the application of *piggyBac* transposon-mediated transgenic techniques in the silkworm and the first successful expression of recombinant human type III procollagen in cocoons of transgenic silkworms^20^,^21^, efforts have been made to explore the silk gland of silkworm to be an efficient system for foreign protein production. Two major expression systems, the fibroin and sericin expression systems, were developed and over ten foreign proteins, including model proteins, human or animal-derived pharmaceutical proteins and silk-based proteins, have been successfully expressed in transgenic silkworm silk glands using these expression systems in the past decade^22^,^23^,^24^,^25^,^26^,^27^,^28^. These results showed that the silk gland is considered to be a cost-effective, easy-scale-up and simply processed bioreactor system for pharmaceutical protein production and silk genetically engineered proteins with improved mechanical properties and new biofunctionalities.

In this study, we successfully produced an r-haFGF protein with high efficiency and biological activity in the cocoons of transgenic silkworm using our previously developed sericin-1 expression system^26^. A strategy involving PIG jumpstarter-induced remobilization of the expression cassette to a “safe harbor” genomic locus for the efficient expression of the transgene was explored and applied to increase the yields of the r-haFGF in the cocoons. The r-haFGF was conveniently purified from cocoons using a simple protocol. Further investigations indicated that the purified r-haFGF provides the same stimulation of NIH/3T3 cell growth, proliferation and *in vitro* wound healing as a commercial haFGF standard. Our results show that the silk gland of silkworm combined with jumpstarter-mediated remobilization could be an efficient bioreactor strategy for the large-scale production of bioactive recombinant haFGF in cocoons.

## Results

### Generation of transgenic silkworm producing the recombinant haFGF proteins in the cocoons

**Figure 1:**
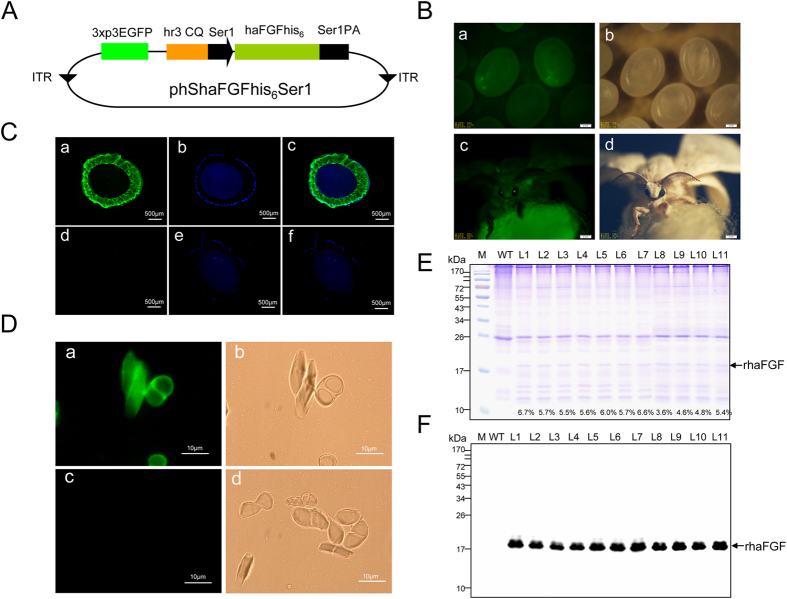
Generation of haFGF transgenic silkworm and the expression analysis of recombinant proteins. (**A**) Structure map of the transgenic vector. (**B**) Fluorescent images of the transgenic silkworm in egg (**a**,**b**) and moth (**c**,**d**) stages, the scale bar represents 2 mm. (**C**) Immunohistochemical analysis of MSG cross sections of transgenic silkworm (**a**–**c**) and non-transgenic silkworm (**d**–**f**). The green fluorescence represents the immunoblot signals of haFGF proteins; the DAPI stained by blue fluorescence represents the cell nucleus. Scale bar represents 500 μm. (**D**) Immunohistochemical analysis of raw silk cross sections of transgenic silkworm (**a**,**b**) and non-transgenic silkworm (**c**,**d**). Scale bar represents 10 μm. (**E**) SDS–PAGE analysis of the cocoon proteins from ten different transgenic lines; the percentages represent the haFGF content of each line in the total cocoon extracts. (**F**) Western blot analysis of the haFGF in cocoon extracts from ten different transgenic lines.

In an earlier report, we constructed the transgenic silkworm, which specifically synthesized recombinant haFGF in the middle silk gland (MSG) of larval silkworm and spun it into the sericin layer of silk using our previously established sericin-1 expression system^26^,^27^. However, the content of haFGF recombinant protein was low and therefore difficult to purify. To improve haFGF production, a more efficient *piggyBac*-based transgenic vector phShaFGFhis6Ser1 was constructed to generate a transgenic silkworm (Fig. 1A,B). Immunohistochemical analysis of the cross sections of the MSG and silk of transgenic silkworm showed that the synthesized haFGF recombinant proteins were secreted into the sericin layer of the MSG lumen, and spun into the sericin layer of silk which was consistent with a previous report^27^ (Fig. 1C,D). Cocoon proteins were analyzed using SDS–PAGE and immunoblotting. A significantly visible protein band on the CBB stained gel with a similar molecular weight of haFGFhis6 was detected and further immunoblotted with an anti-hFGF1 antibody (Fig. 1E,F). The contents of recombinant haFGFhis_6_ in the total cocoon extracted proteins ranged approximately from 3.6% to 6.7% among the 11 transgenic lines (Fig. 1E,F). The different expression levels among the different lines suggested the expression of haFGFhis_6_ might suffer from the chromosome position affect, which had been mentioned in many earlier studies^26^,^29^,^30^,^31^.

### Improving the haFGFhis6 yield in transgenic silkworm by a strategy of **PIG** transposase-mediated genomic remobilization

**Figure 2:**
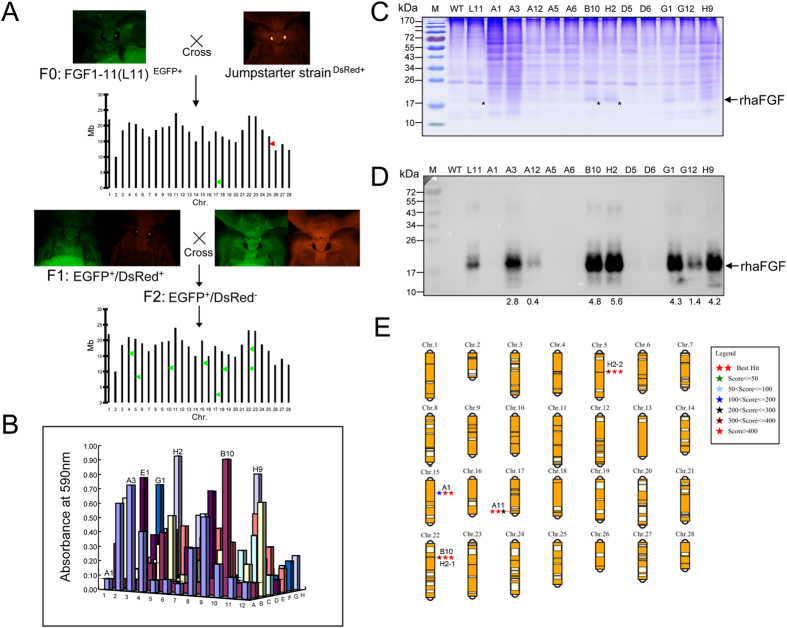
The strategy of PIG transposase-mediated transposon remobilization to improve the expression of haFGF in transgenic silkworm. (**A**) The procedure of the PIG transposase mediated transposon remobilization strategy. (**B**) Large-scale analysis of haFGF expression patterns in individuals of the offspring after hybridization and segregation by ELISA. (**C**) SDS–PAGE analysis of the haFGF proteins in cocoons from typical strains after hybridization with the jumpstarter. The asterisk indicates haFGF proteins. (**D**) Western blot analysis of haFGF proteins in the cocoons of typical strains after hybridization with the jumpstarter. The numbers represent the increased folds of haFGF expression levels in the remobilized mutants compared to that of the A11 line. (**E**) Genetic analysis of the insertion loci of the mutant strains afte r hybridization with the jumpstarter.

Previous studies have shown that transgene expression will suffer host chromosome “position effect” due to the random insertion of the transposon, and higher transgene expression might occur preferably at the safe harbor loci^32^,^33^,^34^. In silkworm, the phenomenon of transgene expression variation caused by the chromosome “position effect” was also observed in many reports^24^,^26^,^29^,^30^,^31^. For higher expression of the transgene, we designed a jumpstart strategy of a piggyBac transposase-induced transposon remobilization to select the potential “safe harbor” loci in the silkworm genome, which permitted a higher expression of the exogenous gene (Fig. 2A). The original haFGF line 11 (L11) with a single transgene copy was hybridized with our previously constructed jumpstarter strain, which stably and universally expressed the piggyBac transposase^35^; the F1 moths with both 3xp3EGFP and 3xp3DsRed markers were then backcrossed with the wild type for segregation of the transposase. An ELISA assay of 96 randomly selected F2 progenies with only the 3xp3EGFP marker showed that the patterns of haFGF expression varied dramatically among those individuals, among which some significantly increased or decreased expression compared to the original L11 line (Fig. 2B). SDS–PAGE and western blot assays of the cocoon proteins from typical mutants further confirmed that haFGF expression levels significantly increased 4.2- to 5.6-fold in strains such as H9, G1, B10 and H2, or severely decreased in lines such as A1, A5, A6 and L9, which gave no detection (Fig. 2C,D). Inverse PCR analysis determined whether the PIG jumpstarter induced the remobilization of piggyBac in the genome, which caused a variation of haFGF expression among F2 progenies. The results showed that the original L11 strain contained one transgene insertion which located at nscaf2766: 342874 locus on Chr.17; after hybridization, the transgene remobilized to nscaf2888: 4013240 locus on Chr.15 in the A1 line, nscaf1681: 5840922 locus on Chr.22 in the B10 line, nscaf1681: 5840922 locus on Chr.22 and nscaf2529: 169295 locus on Chr.5 in the H2 line, respectively (Fig. 2E, Supplementary Table S1 and Figure S1). The results suggested that the transposase triggered the remobilization of transgene to another genomic locus in transgenic silkworm, and induced the expression variation of haFGF in the different strains. Genomic sequence analysis further indicated that the transgene insertions where the haFGF expression was increased located in regions containing no or few endogenous genes nearby (50 Kb upstream or downstream), while those insertions where the haFGF expression level was severely decreased located in intergenic regions containing many endogenous genes which might influence the expression of haFGF (Supplementary Figure S1), suggesting that genomic region containing no or few endogenous genes nearby (50 Kb upstream or downstream) could be favorable for exogenous gene expression. In consequence, the B10 line with a single transgene insertion and high-level expression of haFGF, which accounted for 26% of the totally extracted silk proteins and 5.2% of the cocoon shell weight, was maintained for further studies.

### Purification and refolding of haFGF

**Figure 3:**
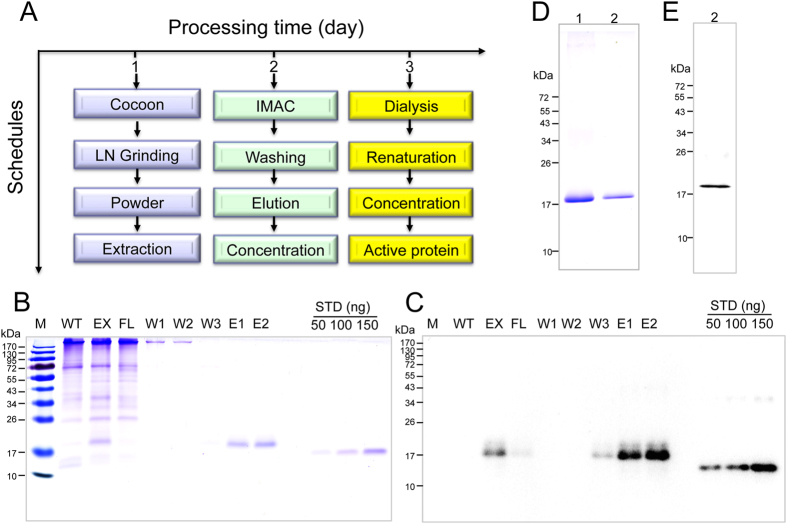
Purification of the haFGF proteins from the cocoons of transgenic silkworm. (**A**) Flow diagram of the processes in purifying haFGF proteins from the cocoons of transgenic silkworms. (**B**,**C**) SDS–PAGE and western blot analysis of the haFGF in each purification process. EX represents the total supernatant extracts of the transgenic cocoon. FL represents the constituents flowing through the Ni charged his-binding column. W1, W2 and W3 represent the three gradient washing steps by 10 mM, 20 mM and 80 mM imidazole, respectively. E1, E2 represent the eluted haFGF by buffer containing 200 mM imidazole or 1 M imidazole, respectively. The yield of the purified haFGF was calculated by comparing the immunoblot band intensity with the FGF1 standard. (**D**,**E**) Analysis of the 200 ng (lane2) and 2 ug (lane1) of pure haFGF by SDS–PAGE and western blot, respectively.

A schedule for the purification of haFGF was created. Processing comprised of extraction and one step of immobilized metal chelated affinity chromatography (IMAC) using a Ni-charged His-binding column, followed by dialysis/refolding and concentration, which takes 3 days (Fig. 3A). In 15 g of cocoon powder from the B10 strain there was an estimated 780 mg of haFGF, after extraction, about a major part of haFGF (~672 mg) could be extracted (Fig. 3B,C, lane 3). The extracted sample was then applied to the Ni-charged His-binding column. SDS–PAGE and western blot showed that the Ni-charged His-binding column effectively separated the haFGF recombinant protein from endogenous silk proteins (Fig. 3B,C, lane 4). Following a gradient washing step, residual silk proteins were further rinsed from the column (Fig. 3B,C, lanes 5–7). The haFGF was then eluted from the column with 200 mM and 1 M imidazole solutions (Fig. 3B,C, lanes 8–9). Finally, approximately 375 mg of haFGF with recovery of 55.8% haFGF could be yielded. Purity of haFGF is more than 95% by calculating the densitometer on the CBB-stained gel (Fig. 3D,E, and Supplementary Figure S2).

### Biological function of the haFGF recombinant protein

**Figure 4:**
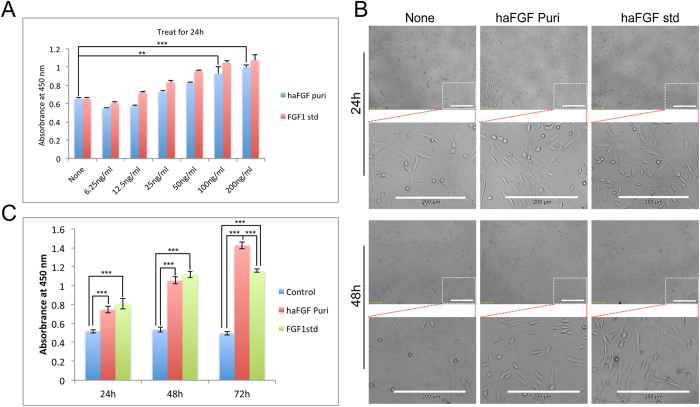
Cell proliferation of purified haFGF on NIH/3T3 cell. (**A**) Cell proliferation assay of NIH/3T3 at 24 h after treating with increasing dosage of growth factors. (**B**) Cell growth observation of NIT/3T3 cells treated with 100 ng/ml of growth factors standard at 24 h and 48 h time sites, respectively. The scale bar represents 200 μm. (**C**) Cell proliferation assay of NIH/3T3 at 24 h, 48 h and 72 h after treating with 100 ng/ml of growth factors. Asterisks indicate statistical significance based on Student’s t-test (*p < 0.05, **p < 0.01, ***p < 0.001).

**Figure 5:**
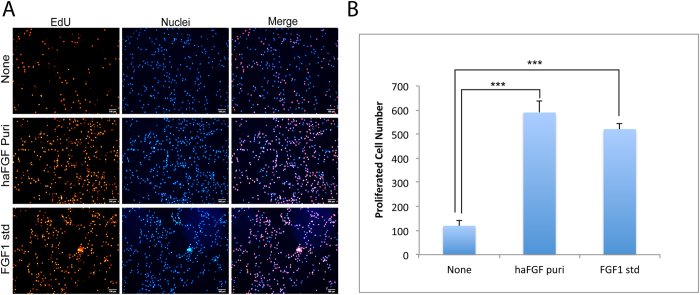
EdU incorporation of NIH/3T3 cells treated with purified haFGF and a haFGF standard compared to controls. Cell nuclei were stained by hoechst 33342 dye. The scale bar represents 100 μm. Asterisks indicate statistical significance based on Student’s t-test (*p < 0.05, **p < 0.01, ***p < 0.001).

**Figure 6:**
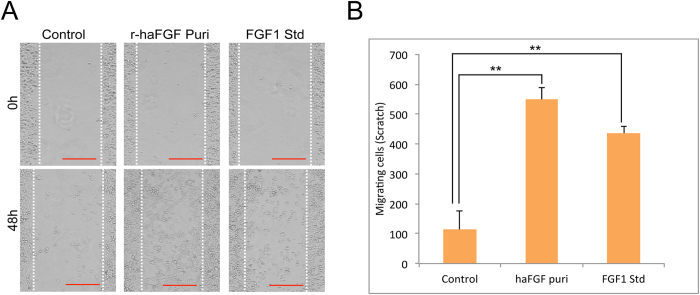
(**A**) *In vitro* wound healing assay by treatment of the scratched NIH/3T3 with purified haFGF and a haFGF standard. Scale bar represents 200 μm. (**B**) The cell numbers in the scratched areas of different groups. Results are representative of three independent experiments. Asterisks indicate statistical significance based on Student’s t-test (*p < 0.05, **p < 0.01, ***p < 0.001).

The bioactivity of the purified haFGF was investigated by cellular experiments. Firstly, the purified haFGF was used to cultivate NIH/3T3 cells and an equal hFGF1 standard was used as the positive control. Cell proliferation of NIH/3T3 could be significantly induced by purified haFGF with dosages of 100 ng/ml or 200 ng/ml (Fig. 4A). Thus the purified haFGF with dosage of 100 ng/ml was used to perform the further assay. The cell growth condition was checked at 24 h and 48 h time sites, respectively. The results showed that the NIH/3T3 cells without haFGF treatment grew poorly, and cellular apoptosis occurred. By contrast, the NIH/3T3 cells treated by purified haFGF or an equal hFGF1 standard grew well; they stretched on dishes and formed the typical morphology of cellular shape (Fig. 4B). NIH/3T3 cells treated with the purified haFGF and an equal hFGF1 standard also showed an increased absorbance at 450 nm at the 24 h, 48 h and 72 h time sites, respectively (Fig. 4C). The cell proliferation effects on NIH/3T3 cells induced by the purified haFGF were slightly lower at the 24 h and 48 h time sites, but higher at the 72 h time site than that of an equal hFGF1 standard, respectively. These results strongly suggested that the purified haFGF recombinant protein showed strong mitogenic activity to promote the cell proliferation of NIH/3T3 cells. Immunocytochemical analysis by EdU incorporation was used to monitor NIH/3T3 cell proliferation; the results showed that cells treated with purified haFGF and an equal hFGF1 standard exhibited strong RFP fluorescence signals comparing to a none-treated control (Fig. 5). A wound-healing assay was performed to investigate the effect of haFGF on NIH/3T3 cell migration. The NIH/3T3 cells treated by purified haFGF and a hFGF1 standard spread into the scratch regions (Fig. 6A), and their migrating cells were significantly higher than that of the control (Fig. 6B), suggesting the purified haFGF promoted chemotactic motility of NIH/3T3 cells and showed equivalent efficacy in wound healing. Furthermore, the economic characteristics of the transgenic silkworm were analyzed. The cocoon and pupa phenotypes of the transgenic silkworm were similar to that of the non-transgenic silkworm, and no obvious difference were found in the weights of the male and female cocoon and pupa between the transgenic silkworm and the wild-type silkworm (Supplementary Figure S3). These results suggest that overexpressing exogenous proteins in silk glands of transgenic silkworms did not influence the economic characteristics of the silkworm, and the silk gland of the transgenic silkworm could be a suitable bioreactor candidate for mass production of bioactive recombinant pharmaceutical proteins.

## Discussion

haFGF is a determinant molecule for a large range of biological processes. It has shown therapeutic potential in wound healing, reconstruction of blood vessels^2^,^3^, cardiovascular disorders^4^,^36^, and the recently discovered insulin resistance and type 2 diabetes treatment^37^ by its strong mitogen activity to stimulate cell proliferation. Over the past decade, increasing demands for haFGF in therapeutic and experimental applications have aroused interest in the cost-effective and efficient production of recombinant haFGF by various expression systems. However, the cost of production of haFGF is still a limitation for its market expansion

The domestic silkworm has the ability to synthesize a large mount of silk proteins in its silk gland and secrete them to make a cocoon, showing potential as a bioreactor for mass production of exogenous recombinant proteins^16^. In this study, we successfully over-expressed haFGF recombinant proteins, with its native bioactivity, in the sericin layer of cocoons by transgenic silkworms. The highly efficient sericin1 expression system, which we previously constructed^26^, was used to regulate the efficient expression of haFGF in the MSG of transgenic silkworm. The haFGF expression cassette carried by a piggyBac-based vector^20^ was integrated into the silkworm genome. The haFGF showed typical expression patterns of endogenous *sericin1* and the synthesized products were secreted into the sericin layer of the MSG lumen, then spun into the sericin layer of silk, in accordance with our previous studies^26^,^27^. The haFGF expression level was analyzed and showed a slight increase compared to that of our previous study^27^, suggesting that changing to a more efficient expression system improved haFGF expression. However, the achieved expression level of haFGF still did not meet the demands for cost-effectiveness and mass production. Thus, more efforts should be made to further improve haFGF expression efficiency.

In the past, major efforts have focused on expression system efficiencies to improve transgene expression to a satisfactory level. However, another critical limitation is chromosome “position affects”^32^,^33^ due to the transposon’s random insertion, which severely suppresses transgene expression. Selecting the line with the highest transgenic expression among the obtained transgenic silkworm lines was the traditional way to deal with the “chromosome position affects”^23^,^24^,^25^. However, this method was inefficient and might be limited by the scale of transgenic silkworm lines. The jumpstarter-mediated enhancer trap had been successfully used to generate mutants with specific expressions of interesting genes in different tissues of the silkworm and other insects mainly through the remobilization of piggyBac to the potential loci of the host genome^38^,^39^,^40^. Thus, the remobilization of the transposon in the genome of the silkworm might be an efficient way to obtain strains with different insertions, offering an opportunity for screening the strains with transgenic expression as high as possible. For that purpose, a previously generated transgenic silkworm which continuously expressed the piggyBac transposase was used as the jumpstarter strain; its average frequency of transposition was estimated to be 61.5%^35^. This jumpstarter strain was hybridized with the original haFGF line and successfully induced the piggyBac remobilization to another chromosome locus of the silkworm. We used ELISA to intensively investigate the haFGF expression patterns of a large mount of the F2 individuals. Most of the expression patterns of haFGF were modest compared to the original haFGF line. Interestingly, several lines among the 96 individuals from the F2 generation showed a dramatically increased expression pattern of haFGF. The insertion loci of these high-expression mutant lines remobilized and distributed in non-coding DNA regions, which were accordingly attributed to be safe regions^34^,^41^. Thus, these loci of the silkworm genome might be considered a “safe harbor” for gene expression. These results show that jumpstarter-mediated piggyBac remobilization was an efficient strategy to obtain mutators with a high expression of exogenous genes.

Benefiting from the convenient expression of the haFGF in a “safe harbor”, the production of the haFGF protein in B10 strains increased by almost five folds, which accounted for 5.2% of the cocoon weight. As a consequence, the recombinant proteins were highly purified with a recovery of 55.8% from the cocoon extract of the B10 strain by a simple method consisting of affinity chromatography and dialysis. After considering that the extraction buffer, containing a high concentration of urea, might denature the activity of haFGF, the purified proteins were subjected to a refolding process with a glutathione redox system following the procedure of a previous study^22^. The refolded haFGF proteins showed the equivalent cell growth promotion activity of a commercial haFGF standard, whereas the purified haFGF without the glutathione redox system did not show such activity at all (data not shown), suggesting the glutathione redox system efficiently refolded most of the haFGF into the correct conformation. EdU incorporation and CCK-8 assays showed that the refolded haFGF possessed the strongest mitogenic activity to promote cell proliferation of NIH/3T3 cells. The refolded haFGF also promoted the cell migration of NIH/3T3 cells, suggesting a potential for haFGF in wound healing applications. We calculated that one transgenic silkworm could produce approximately 2.6 mg of the haFGF recombinant proteins in its cocoon (an average weight of 50 mg). Thus, 2.6 g of the haFGF recombinant proteins could be produced if 1000 B10 silkworms were reared. In conclusion, the results in this study provided the experimental evidence for the feasibility of using transgenic silkworms in the mass production of bioactive haFGF on an industrial scale.

## Methods

### Cell lines and silkworm strains

The NIH/3T3 cell line derived from mouse embryonic fibroblasts was cultivated in Dulbecco’s modified Eagle’s medium (DMEM, Gibco) containing 10% (v/v) fetal bovine serum (FBS, Gibco) at 37 °C in a 5% CO_2_ atmosphere. The Dazao silkworm strain was used as a host to generate transgenic silkworm.

### Vector construction

The haFGF-his6 gene was optimized according to the silkworm codon bias and synthesized commercially. It was then inserted into our previously constructed *Ser1* expression vector pSL1180 [hSer1spDsRedSer1PA]^26^ between *Bam*HI and *Not*I sites to replace *DsRed*. The open reading frame (ORF) containing the target gene was inserted into the basic transgenic vector pBac[3xp3EGFP, af]^42^ at *Asc*I sites to generate the final transgenic vector phShFGF1Ser1PA.

### Generation of transgenic silkworm

The plasmid phShFGF1Sv40 was purified with a plasmid Mini kit (Qiagen) and mixed with phsp70PIG helper at a 1:1 mole ratio^35^ and then microinjected (Eppendorf, Germany) into preblastoderm embryos of the silkworm according to a previously reported method^20^. Hatched G0 larvae were bred to oviposit the G1 eggs, which were fluorescently screened at the body pigmentation stage for EGFP expression in the eyes using an Olympus SZX12 fluorescence stereomicroscope (Olympus). The G1 positive individuals were reared and backcrossed with WT Dazao silkworms to generate stable transgenic silkworms.

### Histological examination

Histological examinations were prepared as previously described^27^. Briefly, the middle silk gland (MSG) of silkworms at the stages of 5^th^ instar day 6 and cocoon shell were fixed overnight with 10% (v/v) formalin, frozen in Tissue-Tek^®^ O.C.T.™ compound (Sakura Finetechnical Co., Ltd.) and crosscut into 10 μm thick sections with a freezing microtome. The sections were then immune-blotted with rabbit anti-FGF1 polyclonal antibody (BioVision), detected with FITC-labeled goat anti-rabbit IgG (Beyotime) and observed under a fluorescence microscope (Nikon).

### Hybridization with PIG jumpstarter

The original haFGF transgenic line (3xp3-EGFP) with a single transgene copy was selected and hybridized with the previously constructed PIG jumpstarter line (3xp3-DsRed)^35^ to oviposit their F1 eggs. The F1 moth with two marker genes (3xp3-EGFP and 3xp3-DsRed) in their eyes were selected and backcrossed to oviposit F2 eggs. To segregate the haFGF transgenic line from the PIG jumpstarter line, the positive moth individuals of F2 that were only expressing EGFP in their eyes were screened and their cocoons and genomes were further subjected to protein and genetic analysis, respectively.

### Genetic analysis-Inverse PCR

Moth genomic DNA was extracted and 20 μg was digested with *Hae*III at 37 °C overnight, purified using the phenol/chloroform method and then 2 μg was circularized by ligation using T4 DNA ligase at 16 °C overnight. The ligated DNA (50–100 ng) was amplified using Taq polymerase under standard conditions with primers Reverse-pBac-F: 5′-TACGCATGATTATCTTTAACGTA-3′ and Reverse-pBac-R: 5′-GTACTGTCATCTGATGTACCAGG-3′ designed from the arm region of the piggyBac vector. Amplified products were separated using agarose gel electrophoresis and recycled using an OMEGA Gel Extraction Kit, then inserted in to the TA-clone vector (TAKARA) for sequencing. The sequences of transgene insertions were analyzed according to the silkworm genome database: SilkDB (http://www.silkdb.org/silkdb/).

### Protein analysis

Cocoons were frozen in liquid nitrogen and immediately shattered into powder. Then, they were immersed into a solution containing 50 mM Tris-HCl, 8 M urea, pH 7.0 at a final concentration of 30 mg/ml overnight at 4 °C to extract the silk proteins. The supernatant samples were collected by centrifugation at 12,000 rpm for 15 min. The concentration of each protein sample extracted from the cocoons was quantified into 1 mg/ml using a BCA Protein Assay Kit (Beyotime). For ELISA analysis, 5 μl supernatant samples were coated in 96-well plates, incubated with anti-hFGF1 antibody (Abcam) in a dilution of 1:10000 and horse radish peroxidase (HRP) labeled goat anti-rabbit IgG (Beyotime) in a dilution of 1:20000, respectively, and visualized by the tetramethylbenzidine TMB regent (Beyotime), then absorbance was measured at 590 nm on the Glomax Multi Detection System (Promega). The measurement was performed in triplicate and repeated independently three times. For SDS–PAGE, 20 μl supernatant samples were separated on 15% (w/v) polyacrylamide gel and stained with coomassie brilliant blue (CBB) R250. For immunoblotting, 5 μl of each sample together with a hFGF1 standard (Biovision) were separated on 15% (w/v) polyacrylamide gel and transferred electrophoretically onto a polyvinylidene fluoride (PVDF) membrane, immunoreacted with anti-hFGF1 antibody (Abcam) in a dilution of 1:10000 and horse radish peroxidase (HRP)-labeled goat anti-rabbit IgG (Beyotime) in a dilution of 1:20000, respectively, and visualized using ECL plus (Amersham Biosciences). The images were recorded using a Chemiscope Series (Clinx Science Instruments). The content of recombinant haFGF was calculated by densitometric measurements of the protein bands on the stained gel or immunoblot using band scan 5.0.

### haFGF purification and refolding

The transgenic cocoons were ground and extracted (25 mg/ml) with extraction buffer (50 mM Tris-cl, pH 7.0, 8 M urea, 250 mM NaCl) over night at 4 °C. Then, the crude extract was filtrated with 5 μm and then 0.5 μm positive pressure filters, respectively. Subsequently, the extraction sample was applied to a Ni SepharoseTM Fast Flow (GE Healthcare) column, following gradient washing steps using wash buffers (50 mM Tris-cl, pH 7.0, 8 M urea, 250 mM NaCl) containing 10 mM, 20 mM and 80 mM imidazole, respectively. The haFGF was then eluted with elution buffer (50 mM Tris-cl, pH 7.0, 8 M urea, 250 mM NaCl, 200 mM imidazole or 1 M imidazole). The eluent products were subjected to SDS–PAGE and western blot analysis as described above or protein was refolded according to a previous report^22^. Briefly, the eluent products were firstly dialyzed (cellulose dialysis membranes, MWCO 1000 Da, Spectrum Laboratory, Inc, USA) in dialysis solution (8 M urea, 1 mM dithiothreitol (DTT), 50 mM Tris–Cl (pH 7.0), and 250 mM NaCl) at 4 °C for 12 h. Then, a half volume of the dialysis solution was replaced with fresh refolding buffer (2.0 mM reduced glutathione (GSH), 0.2 mM oxidized glutathione (GSSG), 1 mM DTT, 50 mM Tris–Cl (pH 7.0), and 250 mM NaCl) every 2 h, four times, to refold the purified haFGF protein and dilute the urea. Finally the eluent products were dialyzed against 50 mM Tris–Cl (pH 7.0) and 200 mM NaCl at 4 °C for 36 h to completely discard the refolding regents. The purified haFGF was then concentrated and partially desalted using a 1 kD ultrafiltration filter (Millipore, Billerica, MA, USA). The final protein concentration was determined by western blotting as described above and Coomassie Plus (Bradford) Assay Kit (Thermo Fisher Scientific Inc., Rockford, IL, USA). The purity of recombinant haFGF was calculated by densitometric measurements of the protein bands on the stained gel or immunoblot using Image J software (http://rsb.info.nih.gov/ij/download.html).

### Cell culture with purified haFGF

NIH/3T3 cells of 96% confluence were seeded into 96-well plates at a density of 500 cells per well and starved in 100 μl of DMEM medium containing 0.5% (w/v) FBS for 16 h to inactivate cell proliferation. Then, the purified haFGF and an equal amount of hFGF1 standard (100 ng/ml) were added to the medium for an additional 72 h in the presence of heparin (8 U/ml), respectively.

### Cell proliferation assay

The NIH/3T3 cells incubated by purified haFGF and FGF1 standard at dosage of 100 ng/ml for 24 h were observed and photographed using a microscope (Nikon). Cell proliferation of NIH/3T3 was measured using a Click-iT^®^ EdU Imaging Kit (Invitrogen) and Cell Counting Kit-8 (Beyotime) following the manufacturer’s protocols, respectively. For CCK-8 assays, CCK-8 solution (10 μl per well of a 96-well plate) was added to each well of NIH/3T3 cells treated by different samples, the plate was incubated at 37 °C for 4 h and then the absorbance was measured at 450 nm using a Glomax Multi Detection System (Promega). The measurement was performed in triplicate and repeated independently three times. For EdU incorporation, NIH/3T3 cells after treatment were labeled by EdU at a concentration of 2 μM at 37 °C over light. Then, labeled cells were immediately fixed with 3.7% formaldehyde in PBS and permeabilized by 0.5% Triton^®^ X-100, followed by EdU detection using 10 μM Alexa Fluor^®^ 555 azide and imaged by fluorescence microscopy at 555 nm excitation and 565 nm emission maxima. The proliferated cell numbers were calculated by the image J software. Data are indicated as mean ± S.D. (standard deviation) for n = 3. Statistical analyses were calculated by Student’s t-test.

### *In vitro* wound healing assay

NIH/3T3 cells were grown in 24-well plates to 100% confluence. Cells were serum-starved for 16 h and wounded by scratching the surface of NIH/3T3 with a sterile pipet tip; cellular debris was removed by washing with PBS. Cells were then stimulated with purified haFGF and FGF1 standard at dosage of 100 ng/ml for 48 h. Initial wounding and the movement of the cells in the scratched area were photographically monitored using a microscope (Nikon). The cell number in the scratched area was calculated using Image J software. Results are representative of three independent experiments. Data are indicated as mean ± S.D. (standard deviation) for n = 3. Statistical analyses were calculated by Student’s t-test.

### Statistical Data Analysis

Data are indicated as mean ± standard deviation (S.D.) for n = 3. Statistical analyses were calculated using Student’s *t*-test; *p < 0.05, **p < 0.01, and ***p < 0.001 was considered statistically significant.

## Additional Information

**How to cite this article**: Wang, F. *et al.* Large-scale production of bioactive recombinant human acidic fibroblast growth factor in transgenic silkworm cocoons. *Sci. Rep.* **5**, 16323; doi: 10.1038/srep16323 (2015).

## Supplementary Material

Supplementary Information
