# Supplementary material for: Large-scale production of bioactive recombinant human acidic fibroblast growth factor in transgenic silkworm cocoons
Source: Sci Rep. 2015 Nov 16;5:16323. doi: 10.1038/srep16323 (PMC4644950; doi:10.1038/srep16323)
Supplement: Supplementary Information [file srep16323-s1.pdf]

Supplementary Information for

Large-scale production of bioactive recombinant human acidic fibroblast growth factor in transgenic silkworm cocoons by Feng Wang et al.

Authors: Feng Wang[1,2], Riyuan Wang[1], Yuancheng Wang[1,2], Ping Zhao[1,2], Qingyou Xia[1,2]*

Author affiliations:

[1]State Key Laboratory of Silkworm Genome Biology, Southwest University, Chongqing, China;

[2]College of biotechnology, Southwest University, Chongqing, China;

*Corresponding author: Qingyou Xia

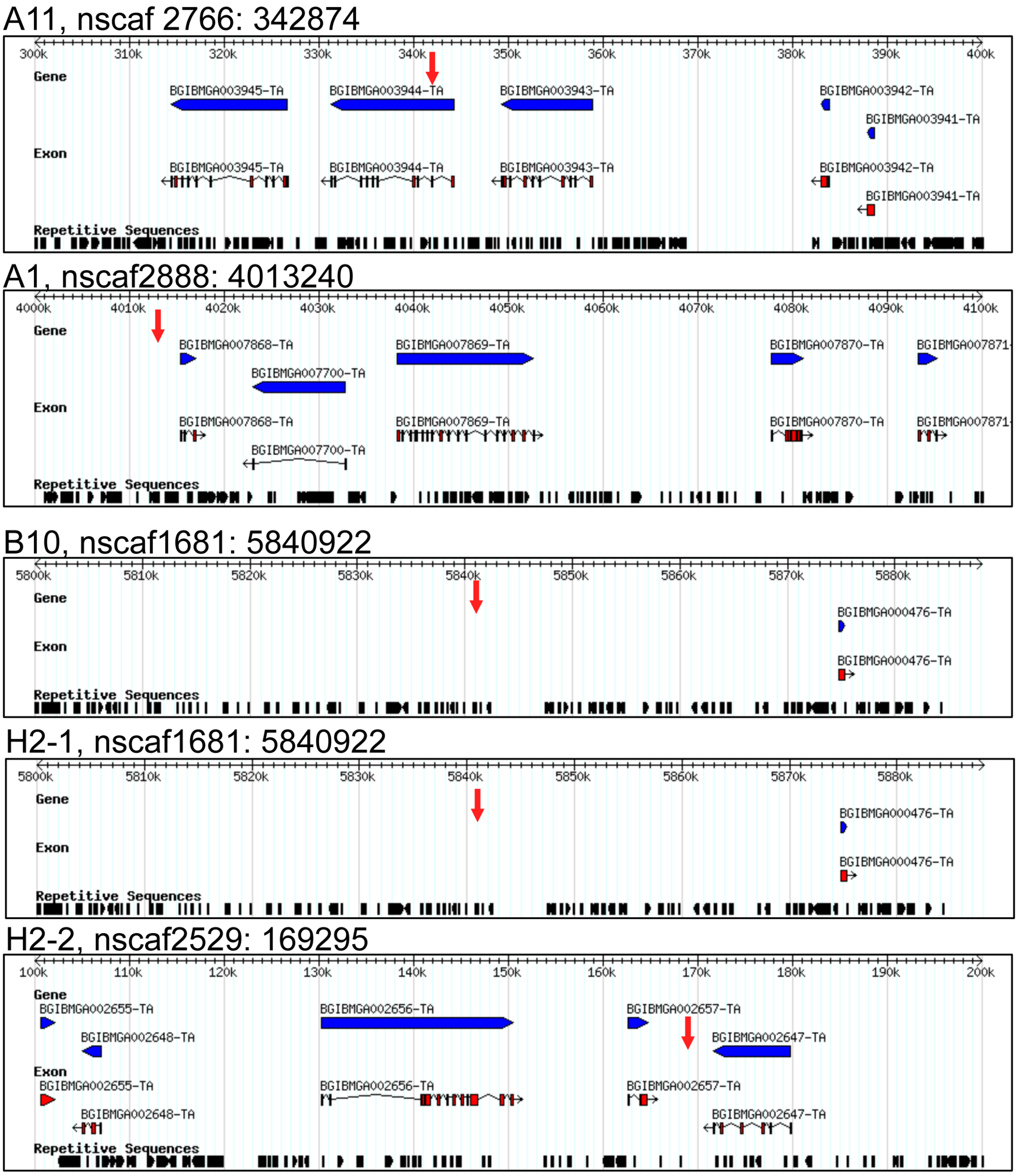

Supplementary Figure S1. Insertion loci information of the mutant strains after hybridization with the jumpstarter. The red arrow represents the insertion loci of each selected mutant strains, and the endogenous genes nearby and their expression orientations were indicated.

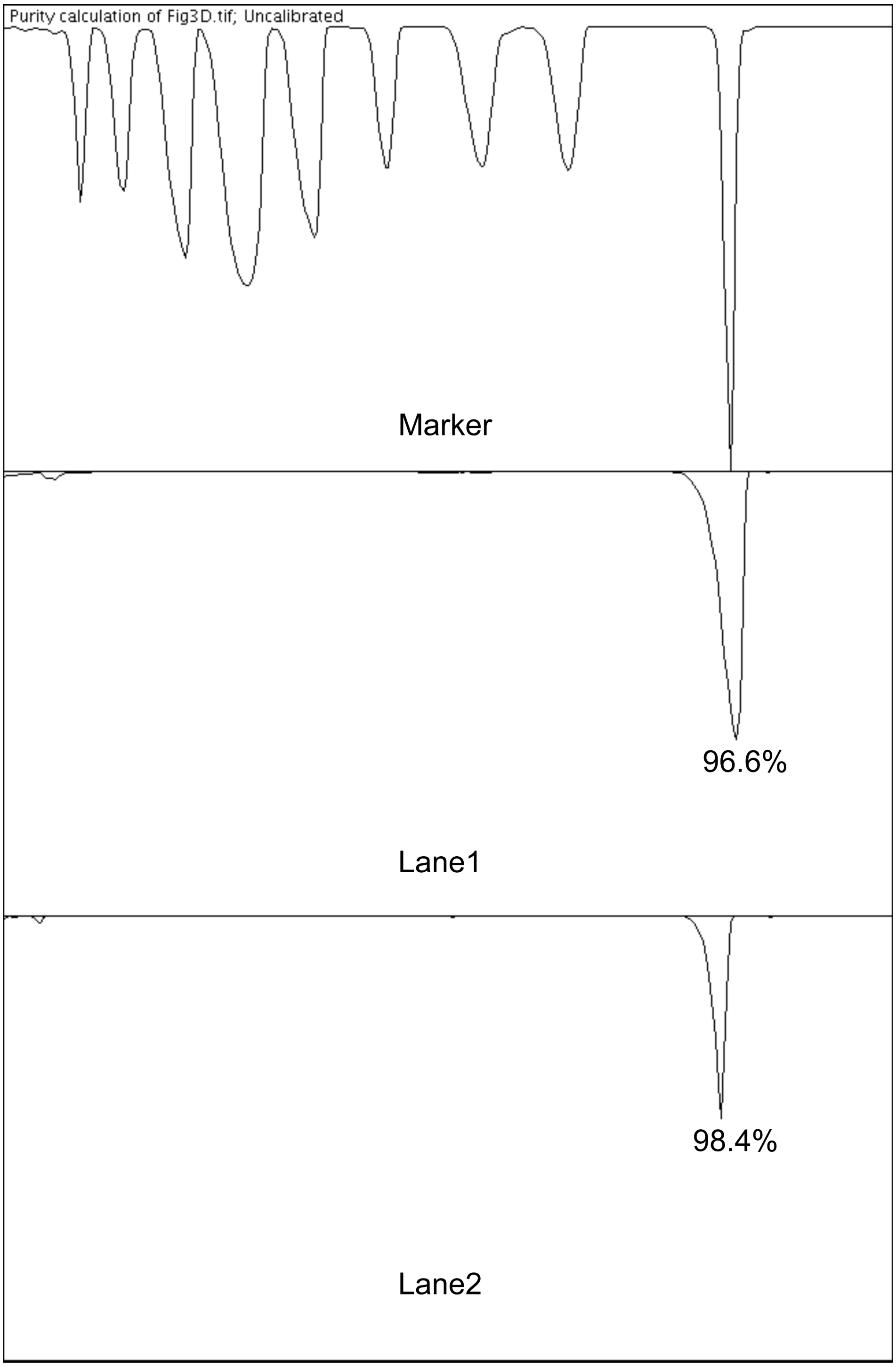

Supplementary Figure S2. Purity calculation of haFGF by image J software.

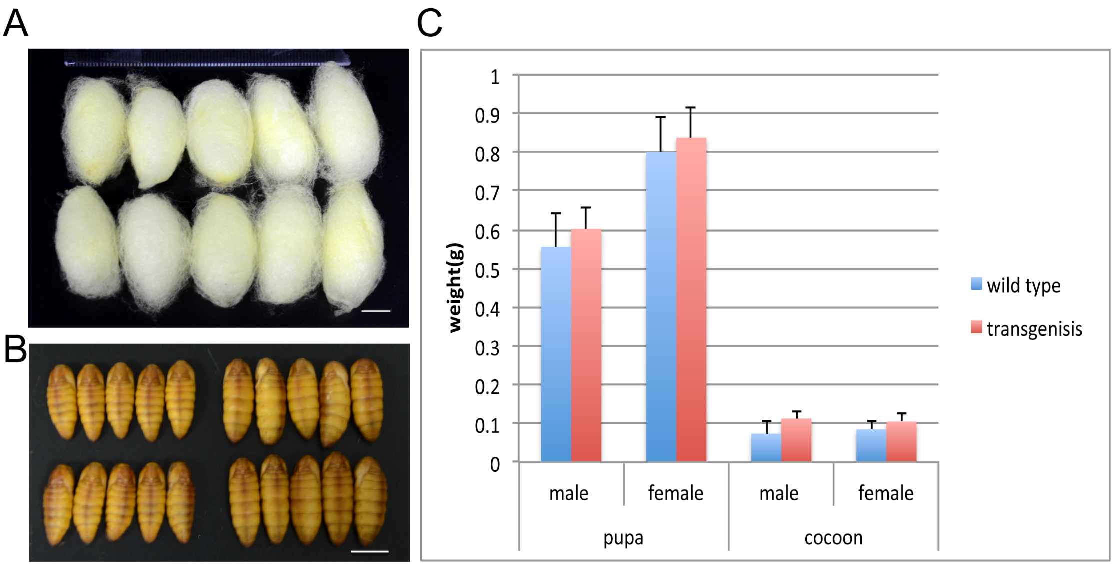

Supplementary Figure S3. Economic characterizes of the transgenic silkworm. A. The morphology comparisons of cocoons between transgenic silkworms (upper) and non-transgenic silkworms (below). B. The morphology comparisons of pupas between transgenic silkworms (upper) and none transgenic silkworms (below). C. Weight statistics of the male and female cocoon and pupa between transgenic silkworm and the wild type silkworm. Statistical data for each group were calculated from 100 individuals.

Supplementary Table S1 integration sites of remobilized transgenes in silkworm genome.

Strain	Chr.	Location	Flanking sequence
A11	17	nscaf2766: 342874	TCAGCTTAGCACTATTATTAA-Transgene-TTAAGGTATGAACTAATAACTT
A1	15	nscaf2888: 4013240	GTAGTAGGGAAACTGATTTAA-Transgene-TTAAGGACAAATAAAATGTTA
B10	22	nscaf1681: 5840922	TGCCAGATTGTTTCCCTTTAA-Transgene-TTAAAAGATCTAAAAAAAAGTC
H2	22	nscaf1681: 5840922	TGCCAGATTGTTTCCCTTTAA-Transgene-TTAAAAGATCTAAAAAAAAGTC
	5	nscaf2529: 169295	CGATCCGAGGCACAATATTAA-Transgene-TTAATGTACTTCAGGTGTACTC
